# The genome sequence of the European water vole, *Arvicola amphibius* Linnaeus 1758

**DOI:** 10.12688/wellcomeopenres.16753.1

**Published:** 2021-06-24

**Authors:** Angus I. Carpenter, Michelle Smith, Craig Corton, Karen Oliver, Jason Skelton, Emma Betteridge, Jale Doulcan, Michael A. Quail, Shane A. McCarthy, Marcela Uliano Da Silva, Kerstin Howe, James Torrance, Jonathan Wood, Sarah Pelan, Ying Sims, Francesca Floriana Tricomi, Richard Challis, Jonathan Threlfall, Daniel Mead, Mark Blaxter

**Affiliations:** 1 Nottingham Trent University, Nottingham, NG1 4FQ, UK; 2 Wellcome Sanger Institute, Wellcome Genome Campus, Hinxton, Cambridge, CB10 1SA, UK; 3 Achilles Therapeutics Plc, London, W6 8PW, UK; 4 Department of Genetics, University of Cambridge, Cambridge, CB2 3EH, UK; 5 EMBL-EBI, Wellcome Genome Campus, Hinxton, Cambridgeshire, CB10 1SD, UK; 6 Owlstone Medical, Cambridge Science Park, Cambridge, CB4 0GJ, UK

**Keywords:** Arvicola amphibius, European water vole, genome sequence, chromosomal

## Abstract

We present a genome assembly from an individual male *Arvicola amphibius* (the European water vole; Chordata; Mammalia; Rodentia; Cricetidae). The genome sequence is 2.30 gigabases in span. The majority of the assembly is scaffolded into 18 chromosomal pseudomolecules, including the X sex chromosome. Gene annotation of this assembly on Ensembl has identified 21,394 protein coding genes.

## Species taxonomy

Eukaryota; Metazoa; Chordata; Craniata; Vertebrata; Euteleostomi; Mammalia; Eutheria; Euarchontoglires; Glires; Rodentia; Myomorpha; Muroidea; Cricetidae; Arvicolinae; Arvicola; *Arvicola amphibius* Linnaeus 1758 (NCBI:txid1047088).

## Introduction

The European water vole, *Arvicola amphibius* Linnaeus 1758, is a small semi-aquatic mammal that lives on the banks of freshwater water courses and in wetlands. *A. amphibius* is native to Europe, west Asia, Russia and Kazakhstan. While the IUCN Red List of Threatened Species reports that *A. amphibius* is of “least concern” worldwide, populations in the United Kingdom have declined to such an extent that the species is considered nationally endangered ( Mathews & Harrower, 2020) owing to habitat loss and predation by the American mink, *Neovison vison*, an invasive alien species. An estimate by Natural England put the 2018 UK population of *A. amphibius* at 132,000, down from 7.3 million in 1990 ( Strachan, 2004). Water voles are absent from Ireland. There have been a number of conservation projects in the UK aimed at supporting populations of *A. amphibius*, including efforts at habitat restoration and to control the population of American mink ( Bryce *et al.*, 2011). There are also efforts to reintroduce the water vole in a number of restored urban and wild habitats. This genome sequence will be of use as a reference for researchers that wish to assess the population genomics of *A. amphibius* and manage reintroductions.

## Genome sequence report

**Figure 1.**
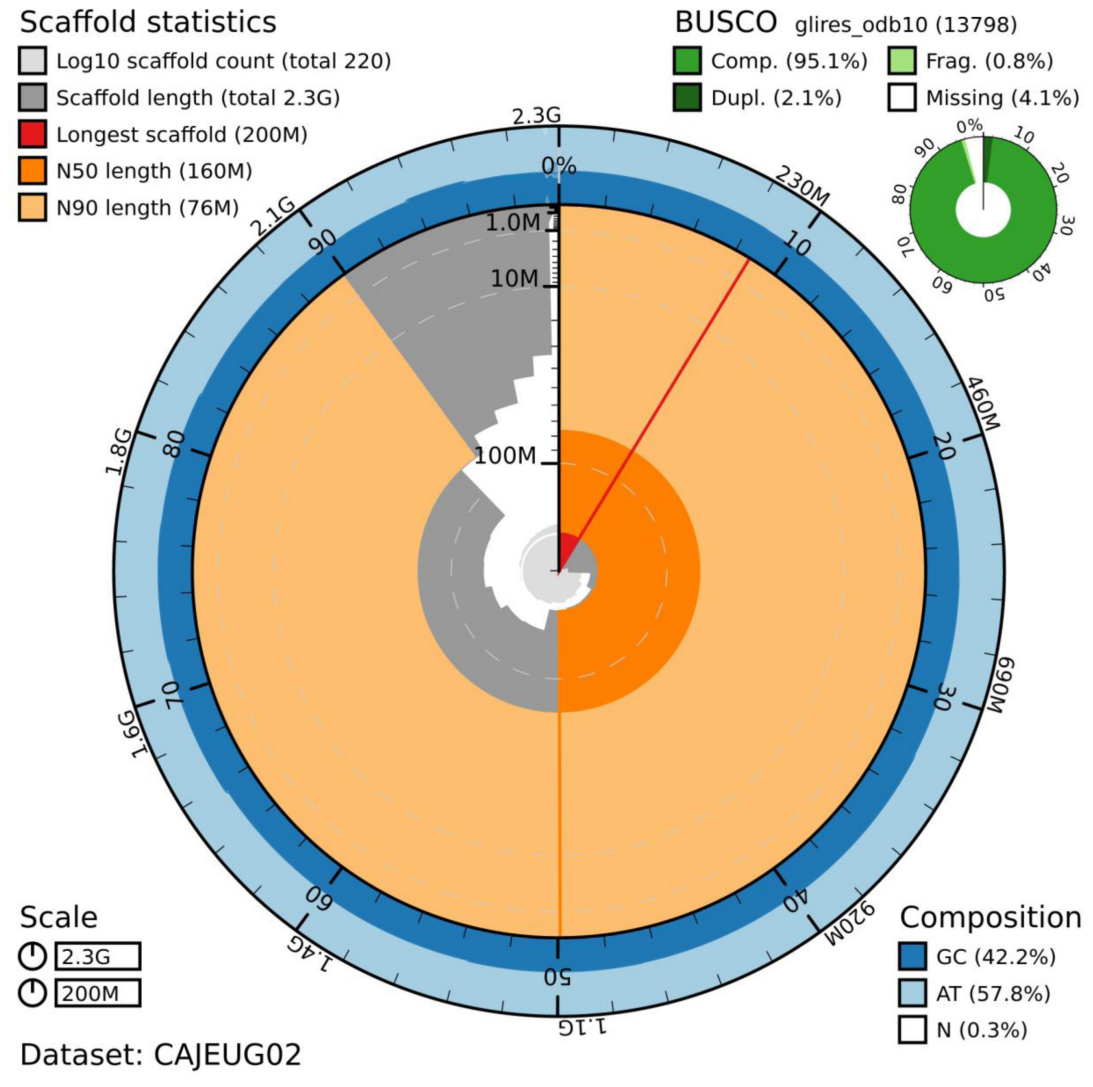
Genome assembly of *Arvicola amphibius*, mArvAmp1.2: metrics. The BlobToolKit Snailplot shows N50 metrics and BUSCO gene completeness. An interactive version of this figure is available at https://blobtoolkit.genomehubs.org/view/Arvicola%20amphibius/dataset/CAJEUG02/snail.

The genome was sequenced from a single male *A. amphibius* collected from the Wildwood Trust, Herne Common, Kent, UK. A total of 45-fold coverage in Pacific Biosciences single-molecule long reads (N50 20 kb) and 52-fold coverage in 10X Genomics read clouds (from molecules with an estimated N50 of 155 kb) were generated. Primary assembly contigs were scaffolded with chromosome conformation Hi-C data. The final assembly has a total length of 2.298 Gb in 216 sequence scaffolds with a scaffold N50 of 138.7 Mb ( Table 1). The majority, 99.4%, of the assembly sequence was assigned to 19 chromosomal-level scaffolds, representing 17 autosomes (numbered by sequence length apart from chromosome 12, which is larger because the previous version of the assembly, mArvAmp1.1, mistakenly labelled this as two separate chromosomes), and the X sex chromosome ( Figure 1– Figure 4; Table 2). The assembly has a BUSCO ( Simao *et al.*, 2015) v5.0.0 completeness of 96.1% using the mammalia_odb10 reference set. While not fully phased, the assembly deposited is of one haplotype. Contigs corresponding to the second haplotype have also been deposited.

**Table 1.** Genome data for *Arvicola amphibius*, mArvAmp1.2.

*Project accession data*	
Assembly identifier	mArvAmp1.2
Species	*Arvicola amphibius*
Specimen	mArvAmp1
NCBI taxonomy ID	txid1047088
BioProject	PRJEB39550
BioSample ID	SAMEA994740
Isolate information	Male; blood sample
*Raw data accessions*	
PacificBiosciences SEQUEL I	ERX3146757-ERX3146763
10X Genomics Illumina	ERX3163119-ERX3163121, ERX3341539-ERX3341546
Hi-C Illumina	ERX3338011, ERX3338012
BioNano	ERZ1392829
*Genome assembly*	
Assembly accession	GCA_903992535.2
*Accession of alternate haplotype*	GCA_903992525.1
Span (Mb)	2,298
Number of contigs	1,085
Contig N50 length (Mb)	5.4
Number of scaffolds	216
Scaffold N50 length (Mb)	138.7
Longest scaffold (Mb)	199.8
BUSCO * genome score	C:96.1%[S:94.1%,D:2.0%],F:0.8%,M:3.1%,n:9226
*Genome annotation*	
Number of protein-coding genes	21,394
Average length of protein-coding gene (bp)	1,700
Average number of exons per gene	11
Average exon size (bp)	208
Average intron size (bp)	4,995

* BUSCO scores based on the mammalia_odb10 BUSCO set using v5.0.0. C= complete [S= single copy, D=duplicated], F=fragmented, M=missing, n=number of orthologues in comparison. A full set of BUSCO scores is available at https://blobtoolkit.genomehubs.org/view/Arvicola%20amphibius/dataset/CAJEUG02/busco.

**Figure 2.**
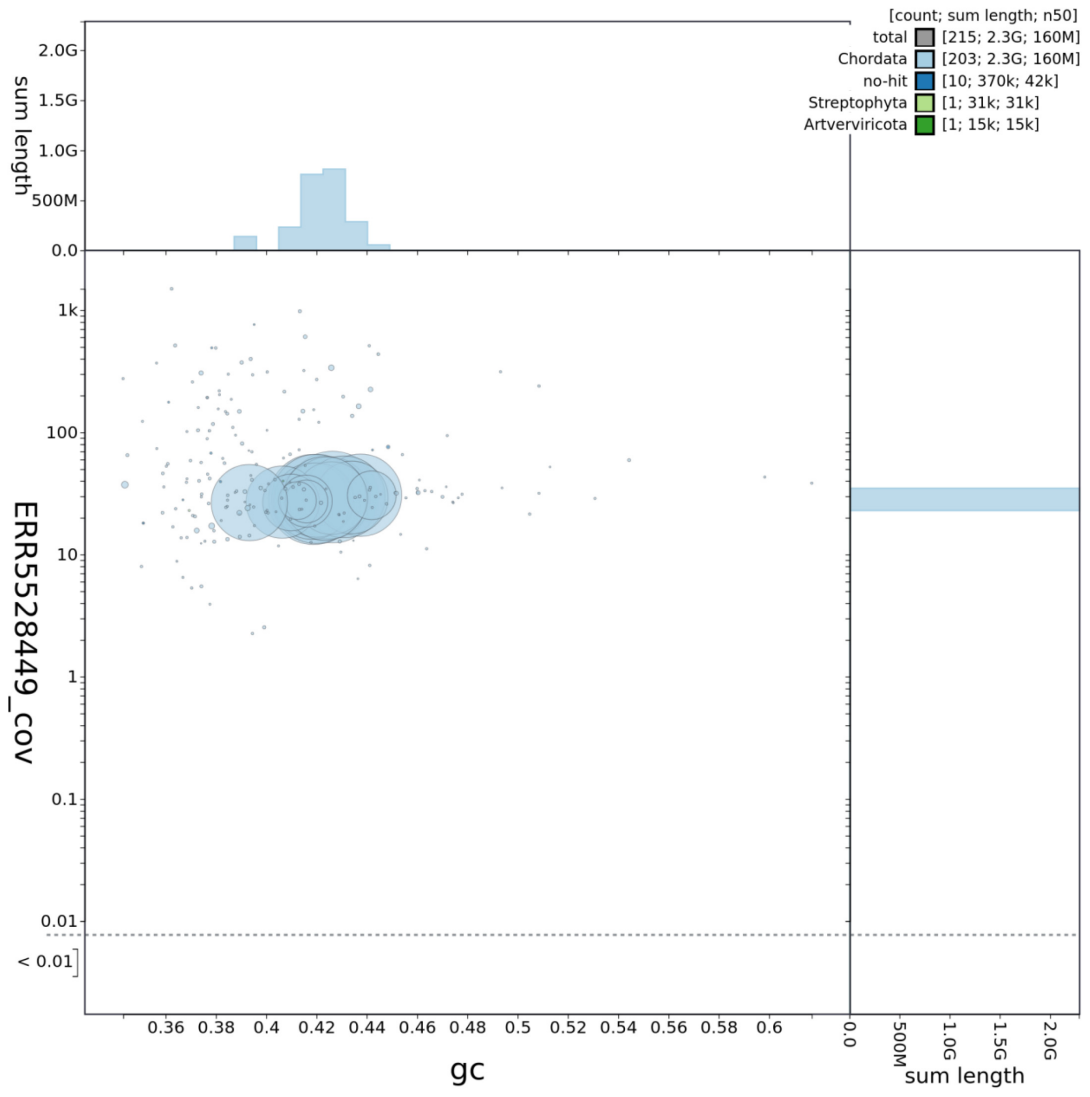
Genome assembly of *Arvicola amphibius*, mArvAmp1.2: GC coverage. BlobToolKit GC-coverage plot. An interactive version of this figure is available at https://blobtoolkit.genomehubs.org/view/Arvicola%20amphibius/dataset/CAJEUG02/blob.

**Figure 3.**
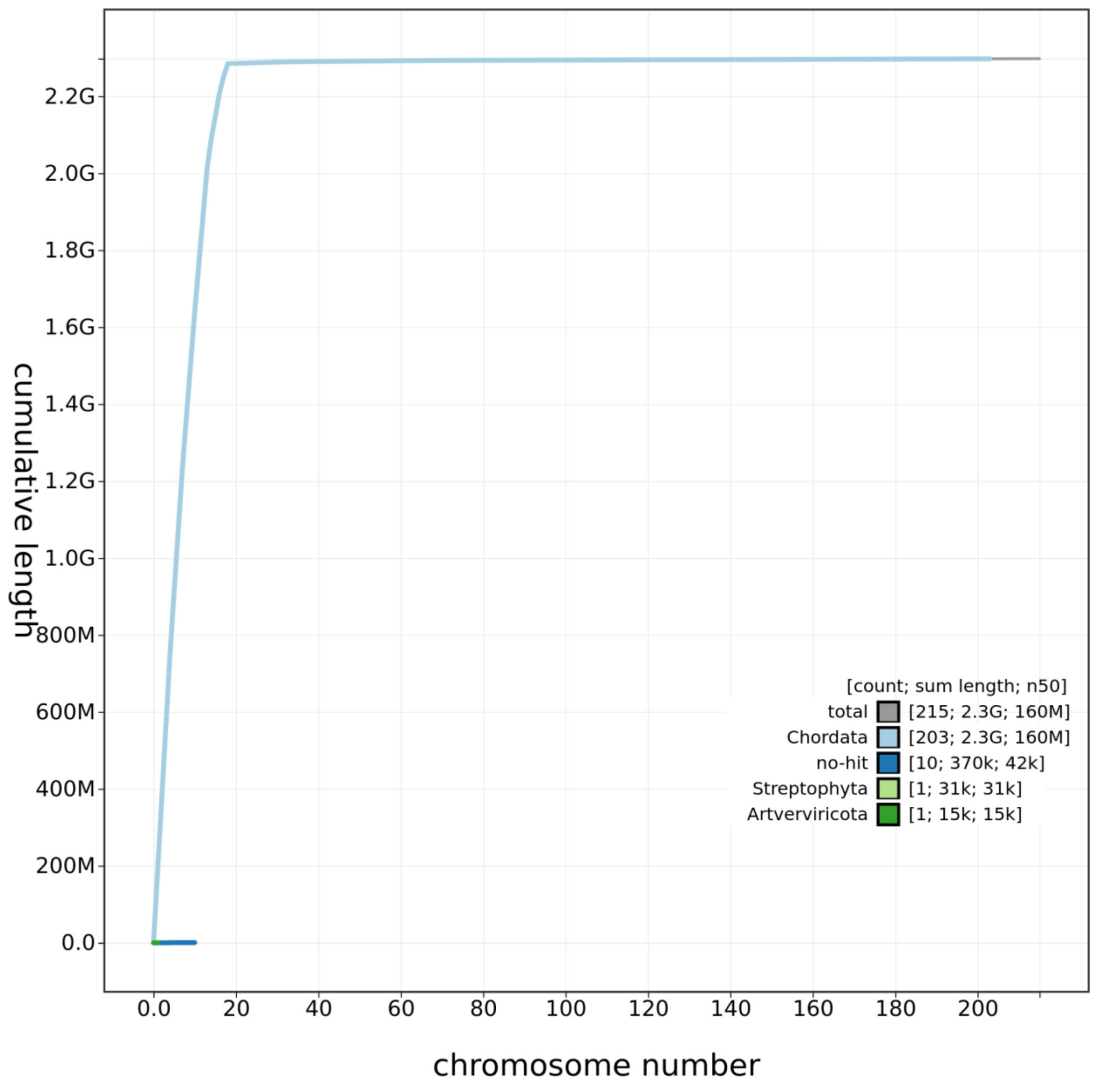
Genome assembly of *Arvicola amphibius*, mArvAmp1.2: cumulative sequence. BlobToolKit cumulative sequence plot. An interactive version of this figure is available at https://blobtoolkit.genomehubs.org/view/Arvicola%20amphibius/dataset/CAJEUG02/cumulative.

**Figure 4.**
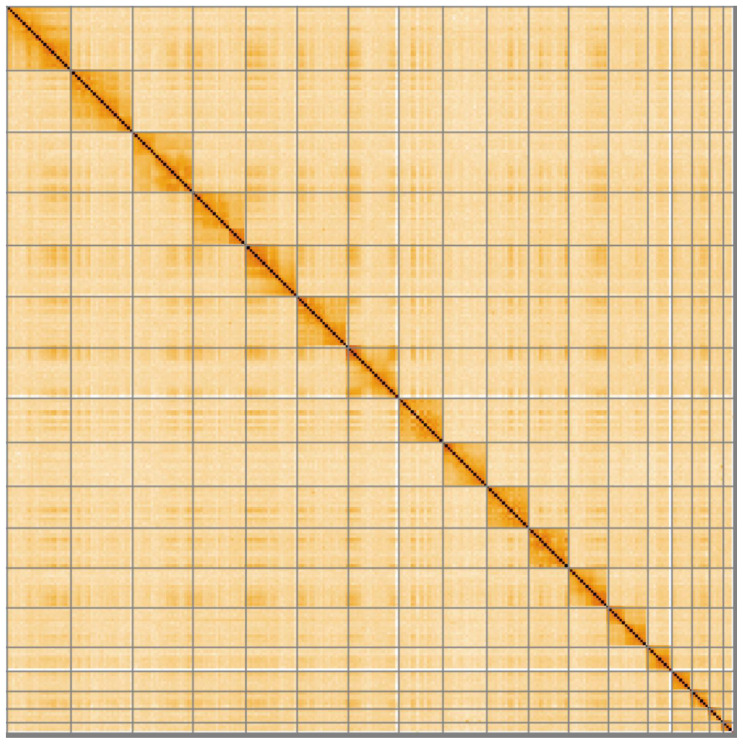
Genome assembly of *Arvicola amphibius*, mArvAmp1.2: Hi-C contact map. Hi-C contact map of the mArvAmp1 assembly, visualised in HiGlass.

**Table 2.** Chromosomal pseudomolecules in the genome assembly of *Arvicola amphibius*, mArvAmp1.2.

INSDC accession	Chromosome	Size (Mb)	GC%
LR862380.1	1	200.53	42.7
LR862381.2	2	193.96	41.9
LR862382.1	3	189.60	42.0
LR862383.1	4	161.33	43.8
LR862384.1	5	160.72	42.6
LR862385.2	6	158.92	43.1
LR862386.1	7	138.66	41.9
LR862388.1	8	131.41	42.0
LR862389.2	9	125.83	43.4
LR862390.1	10	125.09	42.6
LR862391.2	11	123.99	40.7
LR862392.2	12	166.75	42.3
LR862393.1	13	75.71	41.0
LR862394.1	14	63.16	41.6
LR862395.1	15	55.45	44.2
LR862397.2	17	42.65	41.6
LR862398.1	18	33.21	41.2
LR862387.1	X	137.70	39.3

## Gene annotation

The Ensembl gene annotation system ( Aken *et al.*, 2016) was used to generate annotation for an earlier version of the *Arvicola amphibius* assembly ( GCA_903992535.1). Annotation was created primarily through alignment of transcriptomic data to the genome, with gap filling via protein to-genome alignments of a select set of vertebrate proteins from UniProt ( UniProt Consortium, 2019) and coordinate mapping of GENCODE ( Frankish *et al.*, 2019) mouse reference annotations via a pairwise whole genome alignment. The resulting Ensembl annotation includes 34,750 transcripts assigned to 21,394 coding and 2,252 non-coding genes ( Arvicola amphibius - Ensembl Rapid Release).

## Methods

A blood sample was taken from a live male *A. amphibius* specimen that was part of the captive breeding population of Wildwood Trust, Herne Common, Kent, UK (latitude 51.33181, longitude 1.11443). DNA was extracted using an agarose plug extraction from a blood sample following the Bionano Prep Animal Tissue DNA Isolation Soft Tissue Protocol. Pacific Biosciences CLR long read and 10X Genomics read cloud sequencing libraries were constructed according to the manufacturers’ instructions. Sequencing was performed by the Scientific Operations DNA Pipelines at the Wellcome Sanger Institute on Pacific Biosciences SEQUEL I and Illumina HiSeq X instruments. Hi-C data were generated using the Dovetail v1.0 kit and sequenced on HiSeq X. Ultra-high molecular weight DNA was extracted using the Bionano Prep Animal Tissue DNA Isolation Soft Tissue Protocol and assessed by pulsed field gel and Qubit 2 fluorimetry. DNA was labeled for Bionano Genomics optical mapping following the Bionano Prep Direct Label and Stain (DLS) Protocol and run on one Saphyr instrument chip flowcell.

Assembly was carried out following the Vertebrate Genome Project pipeline v1.6 ( Rhie *et al.*, 2020) with Falcon-unzip ( Chin *et al.*, 2016), haplotypic duplication was identified and removed with purge_dups ( Guan *et al.*, 2020) and a first round of scaffolding carried out with 10X Genomics read clouds using scaff10x. Hybrid scaffolding was performed using the BioNano DLE-1 data and BioNano Solve. Scaffolding with Hi-C data ( Rao *et al.*, 2014) was carried out with SALSA2 ( Ghurye *et al.*, 2019). The Hi-C scaffolded assembly was polished with arrow using the PacBio data, then polished with the 10X Genomics Illumina data by aligning to the assembly with longranger align, calling variants with freebayes ( Garrison & Marth, 2012) and applying homozygous non-reference edits using bcftools consensus. Two rounds of the Illumina polishing were applied. The assembly was checked for contamination and corrected using the gEVAL system ( Chow *et al.*, 2016) as described previously ( Howe *et al.*, 2021). Manual curation was performed using evidence from Bionano (using the Bionano Access viewer), using HiGlass and Pretext, and by taking marker data and inspecting 10X barcode overlap using longranger. Figure 1– Figure 3 were generated using BlobToolKit ( Challis *et al.*, 2020). Table 3 contains a list of all software tool versions used, where appropriate.

**Table 3.** Software tools used.

Software tool	Version	Source
Falcon-unzip	falcon-kit 1.8.0	( Chin *et al.*, 2016)
purge_dups	1.2.3-b542dbf	( Guan *et al.*, 2020)
SALSA2	2.2-14-g974589f	( Ghurye *et al.*, 2019)
scaff10x	4.2	https://github.com/wtsi-hpag/Scaff10X
Bionano Solve	3.3_10252018	N/A
arrow	gcpp 1.9.0-SL-release- 8.0.0+1-37-gd7b188d	https://github.com/PacificBiosciences/GenomicConsensus
longranger align	2.2.2	https://support.10xgenomics.com/genome-exome/software/ pipelines/latest/advanced/other-pipelines
freebayes	1.3.1-17-gaa2ace8	( Garrison & Marth, 2012)
bcftools consensus	1.9-78-gb7e4ba9	http://samtools.github.io/bcftools/bcftools.html
gEVAL	N/A	( Chow *et al.*, 2016)
HiGlass	1.11.6	( Kerpedjiev *et al.*, 2018)
PretextView	0.0.4	https://github.com/wtsi-hpag/PretextMap
BlobToolKit	2.5	( Challis *et al.*, 2020)

## Data availability

### Underlying data

European Nucleotide Archive: Arvicola amphibius (European water vole) genome assembly, mArvAmp1. Accession number PRJEB39550. 

The genome sequence is released openly for reuse. The *Arvicola amphibius* genome sequencing initiative is part of the Wellcome Sanger Institute’s “ 25 genomes for 25 years” project. It is also part of the Vertebrate Genome Project  (VGP) ordinal references programme and the Darwin Tree of Life (DToL) project. All raw data and the assembly have been deposited in the ENA. The genome will be annotated and presented through the Ensembl pipeline at the European Bioinformatics Institute. Raw data and assembly accession identifiers are reported in Table 1.
