# Supplementary material for: The genome sequence of the European water vole, Arvicola amphibius Linnaeus 1758
Source: Wellcome Open Res. 2021 Jun 24;6:162. [Version 1] doi: 10.12688/wellcomeopenres.16753.1 (PMC9114827; doi:10.12688/wellcomeopenres.16753.1)
Supplement: Peer review history

# Peer review: Reviewer response for version 1

**DOI:** 10.21956/wellcomeopenres.18475.r50219

**Published:** 2022-05-23

**Authors:** Fahad Alqahtani

**Affiliations:** 1 National Center for Bioinformatics, King Abdulaziz City for Science and Technology, Riyadh, Saudi Arabia

This is an important study, and the authors have sequenced, assembled, and annotated a single male Arvicola amphibious (European water vole). According to the Wildlife Trusts, this species is endangered in Great Britain and on the England Red List for Mammals. From 1990 to 2018, the UK population of the European water vole dropped from 7.3 million to 132 hundred thousand. This population loses about 98% of its species in less than 30 years. Next-generation sequencing technologies are an efficient approach to generating life history and demographic data with respect to the management of endangered wildlife. Here, the authors have used different sequencing technologies (PacificBiosciences SEQUEL I, 10X Genomics Illumina, Hi-C Illumina, and BioNano) and generated a high-quality chromosomal-level assembly of this important species.

 The manuscript is well written and well designed, and the results are clearly presented. The article adds much to the scientific community.

Are sufficient details of methods and materials provided to allow replication by others?

Yes

Is the rationale for creating the dataset(s) clearly described?

Yes

Are the datasets clearly presented in a useable and accessible format?

Yes

Are the protocols appropriate and is the work technically sound?

Yes

Reviewer Expertise:

Applied algorithms in the bioinformatics field. Mitochondrial assembly and Mitochondrial haplogroup assignment.

I confirm that I have read this submission and believe that I have an appropriate level of expertise to confirm that it is of an acceptable scientific standard.

# Peer review: Reviewer response for version 1

**DOI:** 10.21956/wellcomeopenres.18475.r50221

**Published:** 2022-05-17

**Authors:** Alfonso Balmori-de la Puente, Lídia Escoda

**Affiliations:** 1 Institute of Evolutionary Biology (CSIC-UPF), Barcelona, Spain

This manuscript presents the annotated genome sequence of the European water vole ( *Arvicola amphibius*), a small semiaquatic rodent distributed across Europe and Asia. Water vole populations of the genus *Arvicola* have a complex evolution with fossorial and semi-aquatic ecological types (ecotypes), thus this genome sequence can be very convenient to study ecological adaptations in rodents.

 The report is well structured and clearly defined. However, there are some parts in the introduction that need to be better clarified.

 The controversial taxonomic status of this genus, specifically between *A. amphibius* and its sister species *A. scherman*, and the complex genetic structure found in Great Britain is not properly assessed in the introduction. In addition, water voles of the genus *Arvicola* have broad ecological variability that should be better explained. Based on this, the species and the ecotype analyzed should be identified. In addition, the postglacial colonization events of water voles in the United Kingdom might be explained in more detail. All of these aspects will facilitate the future applications of the specimen sequenced for the conservation of the species.

Are sufficient details of methods and materials provided to allow replication by others?

Yes

Is the rationale for creating the dataset(s) clearly described?

Yes

Are the datasets clearly presented in a useable and accessible format?

Yes

Are the protocols appropriate and is the work technically sound?

Yes

Reviewer Expertise:

Conservation Genomics, Ecology and Evolution

We confirm that we have read this submission and believe that we have an appropriate level of expertise to confirm that it is of an acceptable scientific standard.

# Peer review: Reviewer response for version 1

**DOI:** 10.21956/wellcomeopenres.18475.r50220

**Published:** 2022-04-29

**Authors:** Petr Kotlik

**Affiliations:** 1 Laboratory of Molecular Ecology, Institute of Animal Physiology and Genetics of the Czech Academy of Sciences, Liběchov, Czech Republic

This is a short report presenting an annotated complete genome assembly for the water vole, a small mammal species that is widespread in continental Europe but declining and endangered in Britain. The high-quality genome presented here will be an important resource for studies addressing conservation genomics and other questions about the biology of the water vole.

 The manuscript is clearly presented. I have not found any problems in it and therefore have no suggestions for changes.

Are sufficient details of methods and materials provided to allow replication by others?

Yes

Is the rationale for creating the dataset(s) clearly described?

Yes

Are the datasets clearly presented in a useable and accessible format?

Yes

Are the protocols appropriate and is the work technically sound?

Yes

Reviewer Expertise:

Evolutionary biology, population genomics, zoology, vertebrates

I confirm that I have read this submission and believe that I have an appropriate level of expertise to confirm that it is of an acceptable scientific standard.
